# MBD4 Interacts With and Recruits USP7 to Heterochromatic Foci

**DOI:** 10.1002/jcb.25001

**Published:** 2015-01-20

**Authors:** Huan Meng, David J. Harrison, Richard R. Meehan

**Affiliations:** ^1^ MRC Human Genetics UnitInstitute of Genetics and Molecular MedicineUniversity of EdinburghEdinburghUK; ^2^ Key Laboratory of Medical Cell BiologyMinistry of EducationChina Medical UniversityShenyangChina; ^3^ School of MedicineUniversity of St. AndrewsSt. AndrewsUK

**Keywords:** MBD4, UHRF1, USP7, HETEROCHROMATIN REPLICATION AND FORMATION

## Abstract

MBD4 is the only methyl‐CpG binding protein that possesses a C‐terminal glycosylase domain. It has been associated with a number of nuclear pathways including DNA repair, DNA damage response, the initiation of apoptosis, transcriptional repression, and DNA demethylation. However, the precise contribution of MBD4 to these processes in development and relevant diseases remains elusive. We identified UHRF1 and USP7 as two new interaction partners for MBD4. Both UHRF1, a E3 ubiquitin ligase, and USP7, a de‐ubiquinating enzyme, regulate the stability of the DNA maintenance methyltransferase, Dnmt1. The ability of MBD4 to directly interact with and recruit USP7 to chromocenters implicates it as an additional factor that can potentially regulate Dnmt1 activity during cell proliferation. J. Cell. Biochem. 116: 476–485, 2015. © 2014 The Authors. *Journal of Cellular Biochemistry* published by Wiley Periodicals, Inc.

MBD4 is the only methyl‐CpG binding protein that possesses a C‐terminal glycosylase domain. It has been associated with a number of nuclear pathways including DNA repair, the DNA damage response, initiation of apoptosis, transcriptional repression, and DNA demethylation [Bellacosa et al., 1999; Hendrich et al., 1999; Cortellino et al., 2003; Screaton et al., 2003; Rai et al., 2008; Ruzov et al., 2009; Meng et al., 2011; Thillainadesan et al., 2012]. A naturally occurring frameshift mutation in MBD4 results in a truncated protein, lacking its intervening region and glycosylase domain, occurs in human colon and other carcinomas that exhibit microsatellite instability (MSI), generally associated with defects in mismatch repair (MMR) [Bader et al., 1999; Riccio et al., 1999; Bader et al., 2007]. However, mutant mice targeted to create an *Mbd4* null mutation did not exhibit increased tumorigenesis, reduced survival rates, or increased MSI; although a 2–3 fold increase in C:T mutation at CpG sites was observed [Millar et al., 2002; Wong et al., 2002]. Loss of MBD4 function also does not affect MMR‐dependent tumorigenesis [Sansom et al., 2004, 2003], however, it plays a role in mediating the apoptotic response resulting from exposure to DNA damaging agents or inactivation of the maintenance methyltransferase, Dnmt1 [Sansom et al., 2003; Ruzov et al., 2009; Loughery et al., 2011]. Depletion of DNMT1 in cancer cell lines can result in decreases in MMR protein levels, including MBD4, possibly mediated by their physical interaction [Ruzov et al., 2009; Loughery et al., 2011; Laget et al., 2014]. MBD4 strongly associates with heterochromatin [Hendrich and Bird, 1998; Ruzov et al., 2009], and has also been shown to physically interact with and recruit the MMR protein, MLH1 (MutL homolog 1), to heterochromatin sites during MMR‐dependent apoptosis [Bellacosa et al., 1999; Cortellino et al., 2003; Ruzov et al., 2009]. These findings suggest that MBD4 may have additional roles [Cortellino et al., 2003], possibly through unknown protein associations of MBD4 that can contribute to genome stability. Indeed, MBD4 interacting proteins such as Fas‐associated death domain protein (FADD), MLH1, and DNA methyltransferase 1 (DNMT1) potentially link genome surveillance and DNA repair with apoptosis during cell proliferation and DNA replication [Screaton et al., 2003; Ruzov et al., 2009].

UHRF1 (Ubiquitin‐like, with PHD and RING finger domains 1, also known as Np95 and ICBP90) interacts with and recruits DNMT1 to hemi‐methylated DNA to facilitate methylation of daughter strands [Bostick et al., 2007; Sharif et al., 2007]. It also has a binding specificity for 5‐hydroxymethylcytosine (5hmC)‐containing DNA that is similar to its affinity for 5‐methylcytosine DNA [Rajakumara et al., 2011]. UHRF1 is strongly linked to heterochromatin replication and formation [Papait et al., 2008, 2007], which may depend on its specific localization during cell proliferation to the chromocenters (via modified histones) that undergo large‐scale reorganization and progressive clustering at heterochromatin regions [Papait et al., 2008; Nishiyama et al., 2013]. UHRF1 is preferentially expressed in the cells undergoing proliferation [Papait et al., 2008], during which UHRF1 forms a complex with Dnmt1 and USP7 (ubiquitin specific peptidase 7, herpes virus‐associated, also known as HAUSP) [Felle et al., 2011; Qin et al., 2011; Ma et al., 2012]. USP7 regulates the stability of UHRF1 via its deubiquitylase activity [Felle et al., 2011; Qin et al., 2011; Ma et al., 2012], and modulates the enzymatic activity of Dnmt1 on the UHRF1 platform [Bostick et al., 2007; Sharif et al., 2007; Felle et al., 2011; Qin et al., 2011]. However, the nuclear distribution pattern of USP7 per se, is generally diffuse [Holowaty et al., 2003; van der Horst et al., 2006] and is similar to that of MLH1 [Ruzov et al., 2009], which contrasts with the strong heterochromatin and chromocenter‐associated expression of UHRF1 during cell proliferation [Dunican et al., 2013]. USP7 can be recruited and relocated to nuclear foci by its protein partners [Everett et al., 1997; Daubeuf et al., 2009; Zaman et al., 2013], and previous studies have suggested a direct interaction between USP7 and UHRF1 [Felle et al., 2011; Ma et al., 2012]. However, this interaction may be transient and stabilization of the trimeric USP7/UHRF1/DNMT1 complex has been suggested to depend on their mutual engagement on chromatin [Felle et al., 2011].

In this study we performed biochemical isolation and identification of MBD4 associating proteins, and we found that MBD4 specifically interacts with UHRF1 and USP7. We characterized a novel interaction domain in the intervening region of MBD4 and demonstrate a direct role for MBD4 in recruiting USP7 to chromocenters.

## MATERIALS AND METHODS

### PLASMID CONSTRUCTION AND CELL CULTURE

The following plasmids were kindly provided by the laboratories below: SF‐TAP (Dr. Marius Ueffing) [Gloeckner et al., 2007], HA‐UHRF1, FLAG‐UHRF1 (Dr. John Peter McPherson) [Mistry et al., 2008], MCherry‐USP7 (Drs. Heinrich Leonhardt and Fabio Spada) [Qin et al., 2011]. GFP‐MLH1 [Ruzov et al., 2009], FLAG‐p75 (Drs. Madapura M. Pradeepa and Wendy A. Bickmore) [Pradeepa et al., 2012], GFP‐mMBD4 (Dr. Adrian Bird) [Hendrich and Bird, 1998], p21b‐TF‐UB vector (Dr. Il‐Seon Park) [Thapa et al., 2008]. To make FLAG‐hMBD4 (SF‐TAP‐hMBD4), human full‐length MBD4 was cloned to SF‐TAP vector using EcoRI and NotI. To make 6HIS‐TF‐UB‐hMBD4 mutants, the 18‐amino acid (aa) 6‐HIS tag was induced into the upstream of TF‐UB site of the original p21b‐TF‐UB vector, using the QuikChange site‐directed mutagenesis kit (Stratagene). Human MBD4 mutants were then cloned to the p21b‐6his‐TF‐UB vector using BamhI and XhoI. CMT93 and HEK293T were cultured in Dulbecco's Modified Eagle Medium (DMEM; Invitrogen) supplemented with 10% FCS, 1000 U/ml Pen, and 650 µg/ml Strep.

### NUCLEAR EXTRACT PREPARATION AND IMMUNOPRECIPITATION

Nuclear extract was prepared from HEK293T cells according to [Pradeepa et al., 2012]. HEK293T were transfected with SF‐TAP‐hMBD4 and were lysed with a hypotonic lysis buffer (0.05% NP‐40, 10 mM HEPES, 1.5 mM MgCl_2_, 10 mM KCl, 5 mM EDTA, and complete protease inhibitor cocktail (Roche), pH 7.4, 30 × 10^6^ cells/ml). Cytosolic fractions were discarded and the separated cell nuclei were lysed in a nuclear extract buffer (20 mM HEPES, 300 mM NaCl, 20 mM KCl, EDTA‐free complete protease inhibitor cocktail (Roche), pH 7.4, 30 × 10^6^ cells/ml) with or without MNase (Nuclease S7; Roche) as indicated in the result chapter. A final concentration of 5 mM EDTA was used to stop the chromatin digestion if MNae is added, and the sample was centrifuged at 20,000 g for 30 min twice to get post nuclear supernatants. 50 µl sepharose beads covalently conjugated to FLAG‐specific mAb (Sigma) or GFP beads (ChromoTek) were added to samples and incubated for 2 h with rotation at 4°C. Beads were washed three times with ice‐cold nuclear extract buffer containing 0.05% NP40, and once with pure ice‐cold PBS. Bound proteins were eluted by boiling in sample buffer, and the eluted samples were loaded on a large 10% SDS‐PAGE (BioRad) and separated, followed by Western Blot analysis.

### MASS SPECTROMETRY (MS) ANALYSIS

For MS analysis, gels were stained with Colloidal Blue (NuPAGE, Invitrogen). Several chunks of bands of diverse molecular weights were excised from the experimental and control lane of a corresponding molecular weights. The gel chunks were sent to St. Andrews Mass Spectrometry services for analysis. The gel chunk was excised and cut into 1 mm cubes. These were then subjected to in‐gel digestion, using a ProGest Investigator in‐gel digestion robot (Genomic Solutions, Ann Arbor, MI) using standard protocols [Shevchenko et al., 1996]. The MS/MS data file generated was analyzed using the Mascot 2.1 search engine (Matrix Science, London, UK) against the NCBInr database Feb 2011 (12852469 sequences) with no species restriction.

### TRANSIENT TRANSFECTION, RECIPROCAL PULL‐DOWN ASSAY AND WESTERN BLOT ANALYSIS

Lipofectamine 2000 (Invitrogen) was used in accordance with manufacturer's instructions. For reciprocal pull‐down assays, cell nuclear lysates (without chromatin digestion) from 293 T cells that were transiently transfected with plasmids containing FLAG‐UHRF1 or GFP‐Mlh1 were mixed with purified 6HIS‐TF‐UB human MBD4 mutants that were pre‐incubated with Ni‐NTA agarose (Invitrogen) overnight at 4°C on a rotating wheel. After 1 h incubation at 4°C, the mixture containing agarose beads was loaded onto a column, followed by extensive washes (4×) with RIPA buffer followed by pure ice‐cold PBS. The Ni‐NTA agarose was then immediately boiled in loading buffer for western blotting analysis. Western blots were probed with the following primary antibodies: anti‐FLAG antibody (Sigma; mouse F1804, rabbit F7425), anti‐GFP (Roche, mouse 11814460001), anti‐MCherry (Chromotek, RFP antibody [5F8]), anti‐6HIS antibody (gift from Anne Seawright, MRC Human Genetics Unit).

### EXPRESSION AND PURIFICATION OF PROTEINS FROM E. COLI

The respective 6‐HIS‐TF‐UB hMBD4 mutants were transformed into BL21‐CodonPlus®(DE3)‐RIPL cells (Agilent Technologies) in accordance with manufacturer's instructions. Cells were harvested and resuspended in either 20 ml BugBuster® Protein Extraction Reagent (Novagen) with 1 µl/ml Benzonase Nuclease (Novagen), or buffer A (50 mM Na2HPO4/NaH2PO4, pH 7.4, 300 mM NaCl, 10 mM imidazole, 10 mM 2‐mercaptoethanol [2‐ME], 30% glycerol) containing 1 mM 4‐(2‐aminoethyl) benzenesulfonyl fluoride (AEBSF). The cells were then lysed by sonication, or with BugBuster reagent. The soluble fraction was recovered after centrifugation for 30 min at 15,000 g at 4°C. The 6xHis‐tagged proteins were purified by a standard protocol on Nickel agarose (Invitrogen). Proteins were generally eluted with either in a Tris‐ or a phosphate‐based buffer containing 200 mM imidazole, 150–300 mM NaCl, 1 mM beta‐mercaptoethanol, and glycerol if required. After SDS–PAGE analysis to judge yield and purity, the eluted protein was dialyzed against buffer (50 mM Tris‐ or phosphate‐based buffer, pH 8.0, containing 150–300 mM NaCl and 1 mM beta‐mercaptoethanol, and glycerol as necessary) to remove imidazole.

### IMMUNOFLUORESCENCE

The mouse colon cancer cell line, CMT93, was grown on slides and were fixed in 4% paraformaldehyde (pFa) as previously described [Pradeepa et al., 2012]. The primary antibodies were anti‐FLAG antibody (Sigma; mouse F1804, rabbit F7425). Secondary antibodies (Goat α‐rabbit IgG‐Alexafluor Red conjugate [Invitrogen]; Goat α‐mouse IgG‐Alexafluor Green conjugate [Invitrogen]) were diluted 1:1000 in block solution. Coverslips were mounted in ‘Vectashield' mounting media (Vector Laboratories) (250 ng/ml DAPI pre‐mixed). Fluorescence images were taken using a Hamamatsu Orca AG CCD camera (Hamamatsu Photonics), Zeiss Axioplan II fluorescence microscope with Plan‐neofluar objectives and Chroma #83000 triple band pass filter set (Chroma Technology), and ‘IP Lab' software was used for image analysis.

### YEAST TWO‐HYBRID

The Y2H assays were performed as previously described [Dellaire et al., 2002]. The full‐length mouse Mbd4 was cloned into a GAL4 BD‐Bait construct pGBKT7 (Clontech). Yeast strains carrying each plasmid were mated with a strain pretransformed with a mouse embryonic day 11.5 cDNA library cloned into pGADT7 (Clontech). Bait and library clone interaction was identified by β‐Galactosidase assays and appropriate dropout selections and confirmation of restreaking. Confirmed colonies were picked for following colony PCR and plasmid rescue followed by sequencing confirmation.

## RESULTS

### MBD4 FORMS COMPLEXES WITH UHRF1 AND USP7

**Figure 1:**
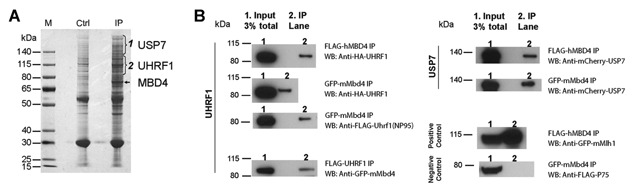
MBD4 complexes with UHRF1 and USP7. (A) UHRF1 and USP7 are identified as new interaction partners of MBD4. MBD4 was immunoprecipitated from nuclear extracts with MNase treatment and subjected to SDS–PAGE and Coomassie blue staining. Protein bands of UHRF1 and USP7 specific for the FLAG‐MBD4‐IP were identified by MS analysis (ctrl: Empty vector). Precipitated MBD4 and the molecular weight marker (M) are indicated. (B) Immunoprecipitation of ectopic proteins with FLAG epitope‐tagged (MNase treated nuclear extract) or GFP epitope‐tagged (no MNase treated nuclear extract) with anti‐FLAG or anti‐GFP antibodies. Co‐precipitated proteins were detected by Western Blot analysis. 3% of the input (Lane 1) and the antibody coupled IP (Lane 2) were loaded. The migration of the molecular weight is indicated on the left.

In order to identify MBD4 interaction partners, we carried out affinity purification of FLAG epitope‐tagged MBD4 from 293 T cells using nuclei extracts prepared with micrococcal nuclease (MNase) digestion (Fig. 1A). We identified UHRF1 and USP7 as two novel interacting proteins by mass spectrometry of the excised regions (Fig. 1A, two brackets). To verify the specific interaction between MBD4 and the two protein partners UHRF1 and USP7, and to clarify if their associations are chromatin‐interaction dependent, we preformed immunoprecipitations (IPs) with FLAG epitope‐tagged human MBD4 in MNase digested nuclear extracts, as well as with GFP epitope‐tagged mouse MBD4 in nuclear extracts without chromatin digestion (Fig. 1B, UHRF1 left & USP7 right). In addition, a reciprocal IP was carried out with FLAG epitope‐tagged human UHRF1 in MNase digested nuclear extracts (Fig. 1B, UHRF1 lower lane). The presence of UHRF1, USP7 as well as MBD4 in a reciprocal IP was tested by Western Blot analysis. MBD4 IP showed co‐precipitation of UHRF1 and USP7 in both conditions with and without chromatin digestion (Fig. 1B, UHRF1 upper two lanes), suggesting these interactions are direct and independent from chromatin binding. Human and mouse MBD4 exhibited similar affinity with UHRF1 (Fig. 1B, UHRF1 upper three lanes) and USP7 (Fig. 1B, USP7), and MBD4 showed a similar interaction by IP with human and mouse versions of UHRF1 (Fig. 1B, UHRF1 upper three lanes), suggesting their interaction is specific and conserved between human and mouse. In addition, a reciprocal IP of UHRF1 precipitated MBD4 (Fig. 1B, UHRF1 lower lane). A co‐IP of MBD4 and Mlh1 confirmed their positive interaction (Fig. 1B, positive control), while the negative control p75 was not precipitated with MBD4 (Fig. 1B, negative control), consistent with the previous reports [Bellacosa et al., 1999; Pradeepa et al., 2012]. Collectively, our data show that MBD4 can interact with UHRF1 and USP7.

### MBD4 INTERACTS WITH UHRF1 COMPLEX THROUGH ITS INTERVENING REGION

**Figure 2:**
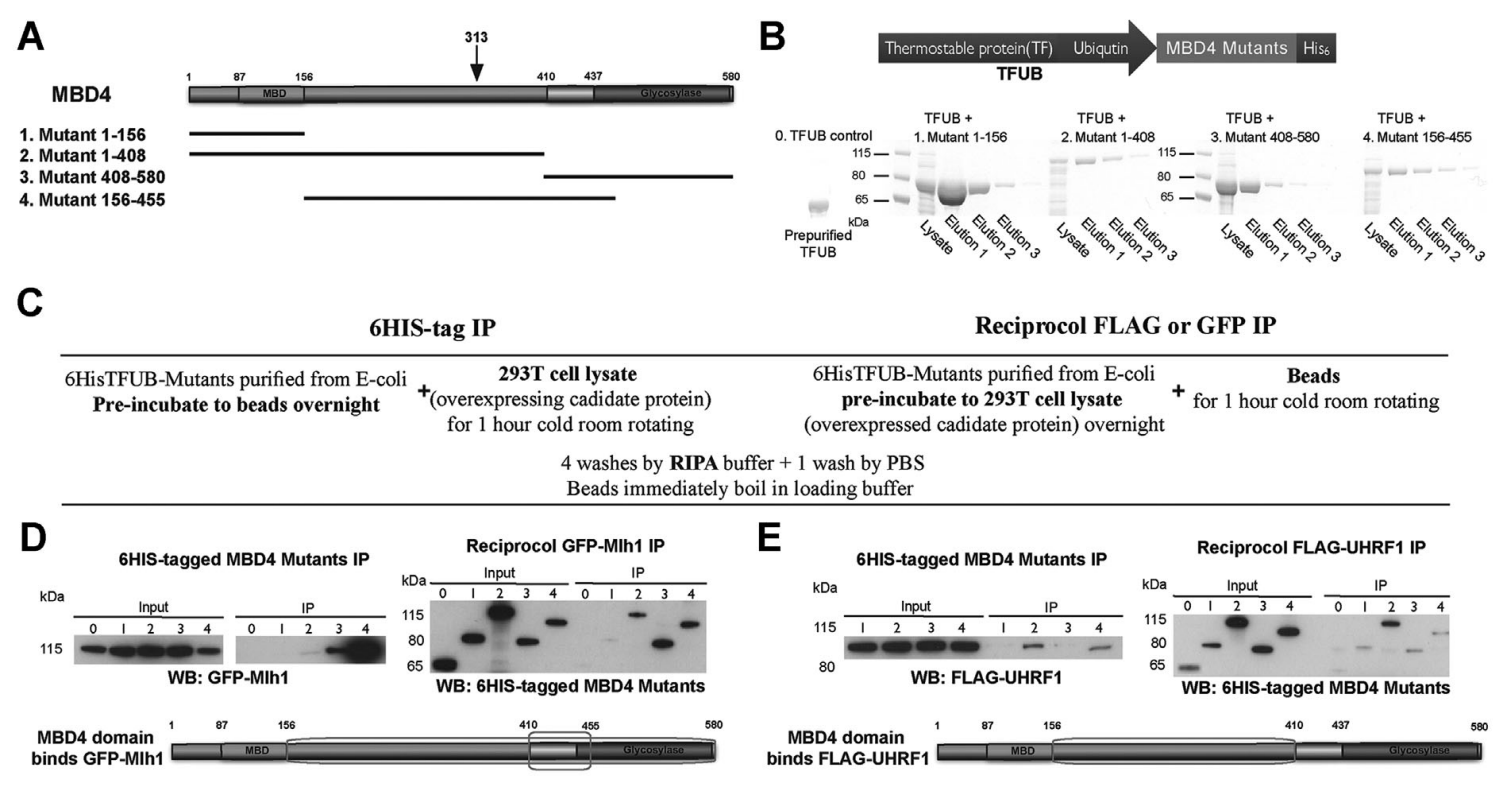
MBD4 interacts with UHRF1 via its intervening region. (A) Schematic representation of the domains of human MBD4 (MBD4 domains are to scale) and the representative scheme of generating the four MBD4 recombinant proteins. A black arrow indicates the natural occurring MBD4 protein truncation site 313 in human MMR‐deficiency carcinomas. (B) The schematic representation of the fusion proteins of MBD4 mutants, and coomaissie staining of the purified MBD4 proteins fused with trigger factor and ubiquitin. Respective total lysates of transformed E.coli cells of MBD4 recombinants 1–4 and their protein elutions are shown as indicated. The dye marker lanes are indicated on the left of lysate lanes with their molecular weight. The empty vector control (labeled as 0. TFUB control) containing TFUB only was from pre‐purified storage, and loaded on the left. The three elutions of individual MBD4 mutants were pooled together and 500 µg of each MBD4 protein mutant was measured for the subsequent reciprocal co‐immunoprecipitation (co‐IP) assays. (C) Flow chart of the reciprocal co‐IP procedure. (D & E) Co‐IP assays to determine the interaction of recombinant MBD4 mutants with Mlh1 (D. control) and UHRF1 (E). Co‐precipitated proteins were detected via western blot. Input (3% of 500 µg GFP‐Mlh1 (D. 6HIS IP) or FLAG‐UHRF1 (E. 6HIS IP), and 3% of 500 µg MBD4 protein mutants in D and E) and the antibody coupled IP are indicated with antibodies used for immunodetection. The migration of the molecular weight is indicated on the left of each blot. The regions labelled by a square on the schematic representation of MBD4 are identified the interacting domains responsible for the associations with Mlh1 (D. control) and UHRF1 (E).

To identify the protein domains of MBD4 mediating the association with UHRF1 complex, we purified the recombinant MBD4 proteins containing the MBD domain (amino acid 1–156), the MBD and the intervening region (amino acid 1–408), the glycosylase domain and its known upstream interaction region (amino acid 408–580), and the intervening region and the downstream interaction region (156–455) (Fig. 2A & B). The four truncated versions of MBD4 were designed to represent the MBD, the intervening region, and the glycosylase domains of MBD4, and to overlap with each other to minimize the interacting regions that may be responsible for partner protein associations (Fig. 2A). These recombinants were 6xHis‐tagged fusion proteins that have a thermostable protein called ‘Trigger Factor' as well as ubiquitin at its C‐terminal region (Fig. 2B, upper), which allows for the isolation of soluble fusion proteins [Thapa et al., 2008]. The four versions of human MBD4 were cloned into the prokaryotic expression vector, induced and purified from *Escherichia coli*; individual protein elutions were collected for the respective mutants (Fig. 2B, lower). Reciprocal in vitro pull‐down experiments were performed using the purified proteins to identify the domains interacting with FLAG epitope‐tagged UHRF1 complex purified from 293 T cells (Fig. 2C). GFP epitope‐tagged Mlh1 was selected as positive control, because the interaction region of MBD4 responsible for its association with Mlh1 was previously identified in a previous yeast two‐hybrid (Y2H) study [Millar, [Link]]. Mutant 3 and particularly mutant 4 of MBD4 efficiently co‐precipitated Mlh1 (Fig. 2D, upper left). An IP with Mlh1 also indicated an interaction with MBD4 recombinants 2, 3, and 4 (Fig. 2D, upper right). These data reveal that the interaction region of MBD4 responsible for the association with Mlh1 resides in the intervening region and glycosylase domain of MBD4 (Fig. 2D, lower cartoon). Amino acids 410–455 corresponds to the overlapping region between mutants 3 and 4 and likely represents the minimum requirement for the association (Fig. 2D, lower cartoon). This was consistent with the previous Y2H study in which amino acid 415–420 of MBD4 was mapped as MBD4 minimum interaction region with Mlh1 [Millar, [Link]]. We then tested the interaction region of MBD4 with UHRF1 (Fig. 2E). Mutants 2 and 4 of MBD4 efficiently co‐precipitated UHRF1 in vitro, while mutants 1 and 3, containing the MBD and glycosylase domain respectively, were not capable of interacting with UHRF1 (Figure 2E, upper left). Reciprocal IP of UHRF1 strongly co‐precipitated mutant 2 of MBD4, by contrast, the other mutants containing MBD and glycosylase domains were very weak (Fig. 2E, upper right). Therefore, we were able to map the interaction region of MBD4 to its intervening region (Fig. 2E, lower). The intervening region of MBD4 does not contain an obvious functional domain [Meng et al., 2011], in addition, our data show that MBD4 recombinant 4 successfully co‐precipitated UHRF1 (similar to the binding of MBD4 recombinant 2) (Fig. 2E, upper left), which contrasts to the very weak binding affinity in the reciprocal assay (Fig. 2E, upper right). This supports the view that the intervening region of MBD4 constitutes a major protein interaction region. The naturally occurring MBD4 truncation at amino acid 313 in MMR‐deficient human carcinomas presumably has the potential to affect the protein interaction profile of MBD4 in addition to losing the catalytically active C‐terminal glycosylase domain (Fig. 2A, black arrow). Taken together, our in vitro studies show that MBD4 can directly interact with UHRF1, and the intervening region of MBD4 can mediate the association.

### MBD4 TIGHTLY COLOCALIZES WITH UHRF1 AT CHROMOCENTERS IN HETEROCHROMATIN REPLICATION AND FORMATION

**Figure 3:**
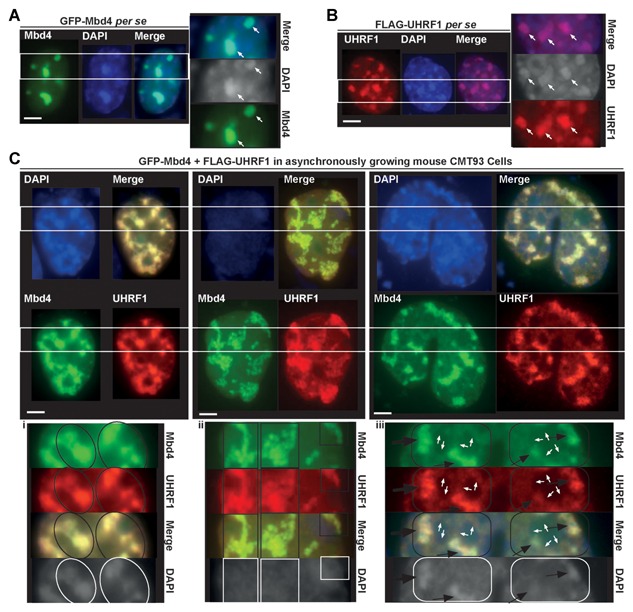
MBD4 tightly co‐localizes with UHRF1 at chromocenters. Asynchronously growing mouse CMT93 cells were grown on coverslips and transfected by GFP‐MBD4 (A), FLAG‐UHRF1 (B), or co‐transfected with GFP‐MBD4 and FLAG‐UHRF1 (C). The cells were fixed 48 h later and analyzed directly by immunofluorescence (IF) (A), or immunostained with anti‐FLAG rabbit primary and goat anti‐rabbit IgG‐Alexafluor Red conjugate secondary antibodies and analyzed (B & C). Nuclear counterstaining was visualized with DAPI. Scale bars, 10 µm. (A) Distribution of GFP‐MBD4 in CMT93 cells. (B) Distribution of FLAG‐UHRF1 in CMT93 cells. (C) GFP‐MBD4 and FLAG‐UHRF1 exclusively colocalized with each other at chromocenters in CMT93 cells. In the immunostaining images in (C. i & ii), the cells exhibit an increase in cell size and marked large‐scale reorganization of heterochromatin, which may be indicative of heterochromatin reformation and replication in interphase. In (C. iii), the cells appear to be undergoing orderly division into two daughter cells. The insets on the right (A & B) or below (C. i, ii, iii) correspond to magnifications of the areas indicated by the two parallel white line. Scale bars, 10 µm.

Previous studies have found that MBD4 and UHRF1 respectively localize to heterochromatic sites in mouse cells [Hendrich and Bird, 1998; Papait et al., 2007; Karagianni et al., 2008; Ruzov et al., 2009; Nady et al., 2011; Dunican et al., 2013; Gelato et al., 2014], suggesting they may be involved in heterochromatin regulation and maintenance. To determine if MBD4 occupies the same cellular space with UHRF1 at heterochromatin, and if their association affects particular cellular phenotypes such as chromatin organization, we performed co‐transfection followed by immunofluorescence (IF) microscopy to determine their potential co‐localization and subcellular distribution (Fig. 3). We used mouse CMT93 cells, a colon cancer cell line that has prominent heterochromatin sites as evidenced by DAPI staining, to test our hypothesis that MBD4 and UHRF1 might associate with each other at heterochromatin sites.

Ectopic GFP epitope‐tagged mouse MBD4 protein exhibited a strong signal that was coincident with DAPI bright spots in the nucleus (Fig. 3A, left & white arrows in right magnification); this resembles the endogenous mouse MBD4 distribution [Hendrich and Bird, 1998; Ruzov et al., 2009]. The DAPI bright spots correspond to methylated satellite DNA, a natural methylated ligand for MBD4 [Ruzov et al., 2009]. Additional non‐heterochromatic staining of ectopic MBD4 was also observed in CMT93 cells (Fig. 3A, MBD4 light green staining). FLAG epitope‐tagged human UHRF1 showed a condensed nuclear distribution that also co‐localized with DAPI bright dots (Fig. 3B, left & right magnification), resembling the location of endogenous UHRF1 protein [Dunican et al., 2013; Gelato et al., 2014]. In CMT93 cells, ectopic expression of either MBD4 or UHRF1 induced minor heterochromatin clustering (Fig. 3A & B, white arrows); a cellular phenomenon previously reported for overexpression of UHRF1 or MeCP2 [Brero et al., 2005; Papait et al., 2008, 2007]. UHRF1 has been demonstrated to regulate cell proliferation [Jenkins et al., 2005], and is expressed at high levels in proliferating cells [Papait et al., 2007]. Consistently and intriguingly, co‐overexpression of UHRF1 and MBD4 resulted in marked large‐scale reorganization events occurring at heterochromatic sites (Fig. 3C, upper & lower magnification), manifested by either bridging (Fig. 3C, left), fragmentation (Fig. 3C, middle), or major clustering (Fig. 3C, right); possibly implying heterochromatin reformation (Fig. 3C, left) or perhaps replication (Fig. 3C, middle) occurs at their co‐localization sites. The cells with such cellular phenotypes are concomitant with a marked increase in cell size (Fig. 3C vs. A & B). In all cases, ectopic MBD4 tightly co‐localized with UHRF1 at chromocenters in CMT93 cells (Fig. 3C i, ii, iii MBD4 vs. UHRF1 and Merge & Supplementary Figure 1B). Specifically, MBD4 and UHRF1 tightly occupied the same cellular space which may perturb chromocenter dynamics during heterochromatin replication and formation (Fig. 3C, lanes of MBD4, UHRF1 & Merge in i, ii & iii, black circle or square). Decondensing heterochromatin or remodeling at chromocenters was manifested by less intense DAPI bright spots, but did not lead to changes in the tight co‐localization of MBD4 and UHRF1 (Fig. 3C, middle, DAPI lane & ii vs. i, iii, DAPI lane, white square or circle). Interestingly, we observed some moderate co‐localization of MBD4 and UHRF1 that may be indicative of dividing cell nuclei (Fig. 3Ciii, white arrows); forming ring‐like clusters that are adjacent to their strong staining at chromocenters (Fig. 3Ciii, black arrows). This may represent an intermediate transition, in which MBD4 and UHRF1 are involved in large‐scale reorganization of heterochromatin (Fig. 3C iii, black arrows in DAPI lane).

### MBD4 DIRECTLY RECRUITS USP7 TO CHROMOCENTERS

**Figure 4:**
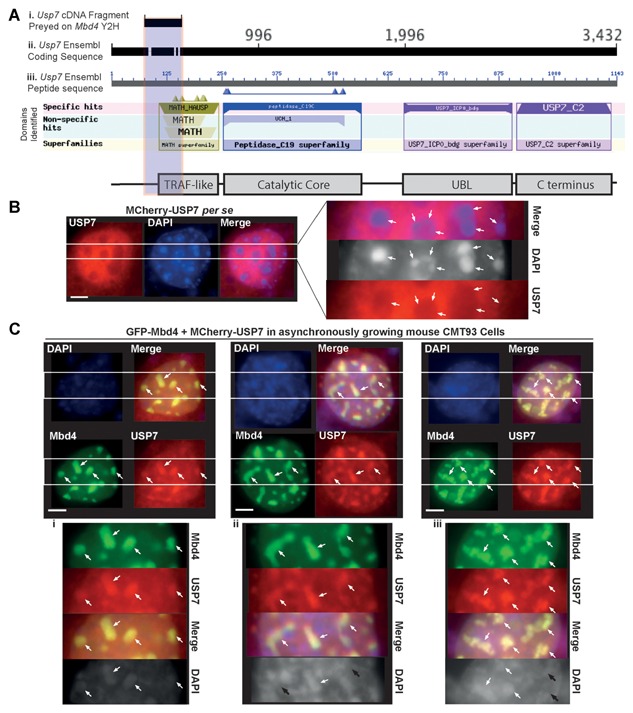
MBD4 directly interacts with and recruits USP7 to chromocenters. (A) The representative scheme for alignment and mapping of a USP7 cDNA fragment that iinteracts with MBD4 in a Y2H screen, (i) USP7 coding sequence, (ii) corresponding peptide sequence, and (iii) schematic representation of domains of USP7. Protein domains are identified by searching Blast integrated SMART domain database (USP7 domains are to scale). The vertical gray shadow indicates the domain region of USP7 overlapping with interacting cDNA fragment. (B & C) Asynchronously growing mouse CMT93 cells were grown on coverslips and transfected by MCherry‐USP7 (B), or co‐transfected by GFP‐MBD4 and MCherry‐USP7 (C). The cells were fixed 48 h later and analyzed directly by IF. Nuclear counterstaining was visualized with DAPI. (B) Distribution of MCherry‐USP7 in CMT93 cells. (C) GFP‐MBD4 recruits MCherry‐USP7 exclusively to chromocenters in all the CMT93 cells co‐transfected. In all the immunostaining images in C, the cells exhibit marked heterochromatin remodeling. The insets on the right (B) or below (C. i, ii, iii) correspond to magnifications of the areas indicated by the two parallel white lines. White arrows indicate triple co‐localization of MBD4, USP7 and DAPI bright spots, while black arrows indicate colocalization of MBD4 and USP7 with diminishing or disappearing DAPI bright spots. Scale bars, 10 µm.

Consistent with our finding that MBD4 directly interacts with USP7, we also identified USP7 as an interactor in a Y2H assay using mouse Mbd4 protein as bait (Fig. 4A), supporting the possibility that the interaction between MBD4 and USP7 is direct. The cDNA fragment of USP7 was aligned to USP7 coding sequence, and it overlaps with C‐terminal TRAF‐like domain of USP7 (Fig. 4A, vertical gray shadow), suggesting that this may be one route through which MBD4 directly interacts with USP7 at the vicinity of the TRAF‐like binding domain of USP7.

Despite different cellular systems and conditions used, previous studies have reported a diffused distribution of USP7 within cell nuclei as well as some cytoplasmic staining [Holowaty et al., 2003; van der Horst et al., 2006]. We determined the subcellular localization of ectopic expression of MCherry epitope‐tagged USP7 in the CMT93 cell model, and our data is consistent with the previously reported nuclear distribution of USP7 as diffused (Fig. 4B, left). More specifically, we characterized that the prominent DAPI bright spots were largely excluded from the nuclear distribution of USP7 (Fig. 4B, left & right magnification, white arrows), implying USP7 may require additional heterochromatin‐associating interaction partner(s) to be recruited to the chromocenters where UHRF1 is tightly bound. Indeed, a number of studies have shown that USP7 can be recruited and relocated to particular nuclear loci via physical interactions with its protein partners, to effectively participate in cellular processes such as DNA damage and repair, apoptosis, and innate immunity response [Everett et al., 1997; Daubeuf et al., 2009; Zaman et al., 2013]. Moreover, MBD4 has been reported to possess a recruitment function by which it leads to the re‐distribution of diffused MLH1 to accumulate at DAPI bright spots that are associated with heterochromatic chromatin in MEF cells [Ruzov et al., 2009], and we have observed the same phenomenon in the CMT93 cell model (Supplementary Figure 1 A). This suggested to us that MBD4 might possess the ability to participate in the UHRF1/Dnmt1/USP7 trimeric complex by facilitating the recruitment of USP7 to the chromocenters. To address this question, we studied the subcellular localization of USP7 in the presence of ectopic MBD4 (Fig. 4C). GFP epitope‐tagged MBD4 was detected exclusively at chromocenters (Fig. 4C, green staining), to which UHRF1 was shown in Fig. 3 to be tightly bound. Strikingly, chromocenter‐binding MBD4 was able to recruit USP7 to chromocenters in all co‐transfected CMT93 cells tested (Fig. 4C, MBD4, USP7 and Merge, white arrows & Supplementary Figure 1 C). In agreement with the above observations of co‐localization of MBD4 and UHRF1 in Fig. 3, the MBD4‐induced USP7 relocation and their co‐localization at chromocenters also leads to a degree of heterochromatin reorganization that was not evident in the single transfections (Fig. 4C, upper, left & right vs. middle DAPI lane & disappearing heterochromatin spots indicated by black arrows in 4 C ii & iii). We observed heterochromatin clustering and diminution (Fig. 4C i DAPI lane, white arrows), chromocenter linkage and fragmentation (Fig. 4C ii DAPI lane, white arrows), and marked heterochromatin remodeling at chromocenters (Fig. 4C iii DAPI lane, white arrows). Collectively, our data show that MBD4 directly interacts with and recruits USP7 to chromocenters during interphase, where MBD4 and UHRF1 can also be tightly co‐localized and this is concomitant with large‐scale reorganization of heterochromatin.

## DISCUSSION

During cell proliferation, UHRF1 may act as a recruitment platform to facilitate faithful inheritance of DNA methylation patterns [Bostick et al., 2007; Sharif et al., 2007]. Mounting evidence indicates that USP7 can regulate the protein stability of UHRF1 as well as Dnmt1 through its deubiquitylase activity [Sharif et al., 2007; Ma et al., 2012]. In this report, we have shown that MBD4 can directly interact with and recruit USP7 to the UHRF1 platform at heterochromatin‐associated chromocenters. Importantly, regulation of the interaction between UHRF1 and deubiquitylase USP7 has been shown to be cell cycle dependent [Ma et al., 2012]; UHRF1 is expressed and protected by USP7 from auto‐ubiquitinylation during G1 and S phases of the cell cycle, and M phase‐specific phosphorylation of UHRF1 expels USP7 from UHRF1 platform [Ma et al., 2012], leading to proteasomal degradation of UHRF1 and perhaps Dnmt1 as well [Felle et al., 2011; Ma et al., 2012]. MBD4 may have a role in this process via its interaction with all three components.

Recent evidence indicates that the MBD4 protein is essential for cell survival following oxidative stress [Laget et al., 2014]. MBD4 and DNMT1 can be recruited at sites of oxidation‐induced DNA damage, where they may participate in DNA repair or cell death pathways [Ruzov et al., 2009; Laget et al., 2014]. Although cell models and physiological conditions are different, our MBD4 interaction data suggest that UHRF1 and USP7 might also participate in these pathways. Our current study provides a foundation for future functional studies of these new MBD4 interactions and roles in related pathways of cell proliferation, stress response or DNA methylation machinery.

The intervening region within MBD4 was previously viewed as a functional desert [Meng et al., 2011], our characterization strongly suggests that this region may possess a significant potential for novel protein interactions, which may augment MBD4's well‐characterized methyl‐CpG binding and glycosylase repair functions [Millar, [Link]; Screaton et al., 2003; Meng et al., 2011]. Motif and primary sequences of the intervening region are poorly conserved between lower and higher vertebrates, suggesting additional protein structure and/or motifs acquired in the latter may attract new functional interactions. This might link to recurrent frameshift mutations in MBD4 found in a number of human cancers exhibiting MMR deficiency resulting in human MBD4 protein truncation at amino acid 313, which would presumably deconstruct the interaction function of intervening region in addition to loss of the glycosylase domain of MBD4.

Recent studies have documented the overexpression of MBD4 partner proteins, UHRF1 and USP7, in a variety of human cancers, which often correlate with a poor outcome [Unoki et al., 2010, 2009; Mudbhary et al., 2014]. In addition, MBD4 activation has been shown to be a consequence of RON overexpression that results in reprogrammed DNA methylation at specific target genes, which is associated with metastasis and poor patient outcomes [Cunha et al., 2014]. Future studies will be required to address if the MBD4 protein interaction with UHRF1, USP7, and the previously identified partner DNMT1 have novel functions with respect to targeting specific methylation patterns at defined loci as well as in maintaining genome‐wide methylation patterns at chromocenters. This interaction may also impact on nuclear organization, chromatin remodeling, and histone modifications in relevant cancers [Jones, 2012; Cunha et al., 2014].

## Supporting information


**Figure S1**: Co‐localization of MBD4 with Mlh1, UHRF1, and recruitment of USP7 at heterochromatic foci.
