# Supplementary figures and images for: MBD4 Interacts With and Recruits USP7 to Heterochromatic Foci
Source: J Cell Biochem. 2015 Jan 20;116(3):476–85. doi: 10.1002/jcb.25001 (PMC4964934; doi:10.1002/jcb.25001)

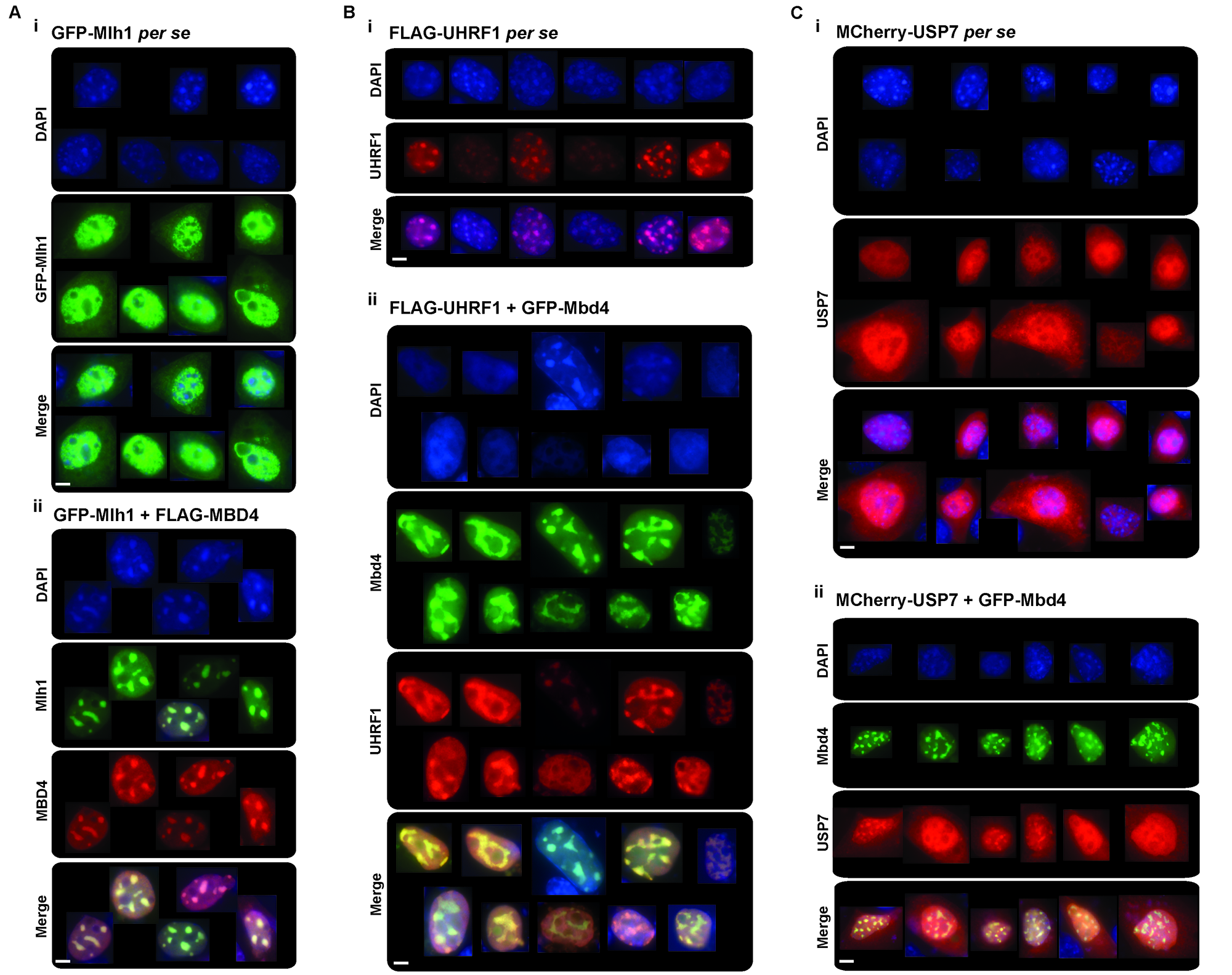

Supplement: Supplementary file 1 — Figure S1: Co‐localization of MBD4 with Mlh1, UHRF1, and recruitment of USP7 at heterochromatic foci. [file JCB-116-476-s001.tif]
